# Supplementary material for: One-pot universal initiation-growth methods from a liquid crystalline block copolymer
Source: Nat Commun. 2019 Jun 3;10:2397. doi: 10.1038/s41467-019-10341-7 (PMC6546696; doi:10.1038/s41467-019-10341-7)
Supplement: Supplementary file 1 — Supplementary Information [file 41467_2019_10341_MOESM1_ESM.pdf]

Supplementary Information for

One-Pot Universal Initiation-Growth Methods from A Liquid Crystalline Block Copolymer

Bixin Jin,[1,2] Koki Sano,[3] Satoshi Aya,[3] Yasuhiro Ishida,[3] Nathan Gianneschi,[4] Yunjun Luo,[1,2]* Xiaoyu Li[1,2]*

*Corresponding author. E-mail: yjluo@bit.edu.cn; xiaoyuli@bit.edu.cn.

Supplementary Methods

High-resolution differential scanning calorimetric (HR-DSC). To study liquid crystalline phase transition of the micellar systems in solution state, we carried out a heat-capacity investigation by using a home-made HR-DSC instrument. The sample solutions were loaded into an aluminum cup and hermetically sealed with an aluminum lid. The mass of samples was fixed to be 18 mg for the measurements. In the measurements, a heat flux type HR-DSC, equipped with semiconducting thermoelectric modules, was used to measure the temperature difference in a sample cell and a reference cell as a function of temperature of DSC chamber as in conventional DSC measurements, providing excellent temperature resolution of <1 mK and baseline stability. Thus, this technique allows us to measure thermal events with a high sensitivity of nano-watt level. Details of principle of the instrument and some applications are described elsewhere[1-3]. All the measurements were performed with a super-slow scan rate of ±0.01 °C/min in order to put the samples as close as possible to their equilibrium state.

Nuclear magnetic resonance (NMR). ^{1}H NMR spectra were recorded using Avance 500 (Bruker A.G.) instruments (operating at 500 MHz) at ambient temperatures. Chemical shifts (*δ*) were determined using tetramethylsilane for ^{1}H NMR as internal reference.

Grazing incident x-ray diffraction measurement (GI-XRD). In order to further explore the involved molecular packing and phase structure, we conducted additional GI-XRD measurement. GI-XRD analysis was performed at 25 ºC using a D8 Discover with GADDS (Bruker A.G.) diffractometer operated at 40 kV and 40 mA with Cu Kα radiation (λ = 0.15406 nm), and the sample-to-detector distance was 10 cm. The GI-XRD image and a plot of the scattering intensity as a function of wave number were included as Supplementary Figure 4c and 4d. The signals at 1.98, 3.96 and 5.97 nm^{-1} at small angles correspond to (001), (002) and (003) lamellar planes. The (001) distance is the interdistance between the lamellar layers. The diffraction signal at 12.13 nm^{-1} at wide angles corresponds to the intermolecular packing distance

perpendicular to the local molecular orientation of mesogenic perfluorooctyl moieties. Since the so-called smectic B type lamellar phase usually has a tight intermolecular packing and forms hexatic ordering within a lamellar plane, a sharp wide-angle signal that overlaps with a broad signal as similar to the one we observed at about 12.13 nm^{-1} would be expected. However, the sharp signal is absent in the present data. Therefore, judging from this perspective, the present liquid crystal phase is more likely a smectic A type lamellar phase, instead of other highly ordered smectic phases. Further detailed analysis could be made by measuring an aligned cylindrical micelle sample in future, which can be effort-consuming and beyond the scope of this manuscript.

Preparation and Characterization of $P2VP_{46}$/PhSeBr complex. To evaluate the efficiency of the dynamic covalent bonding between PhSeBr and pyridyl groups, $P2VP_{46}$ homopolymer (30 mg) and PhSeBr (13.6 mg, 20 % molar of the pyridyl groups) were dissolved in *i*-PrOH. The solution was held at 80 °C for 20 min and cooled to room temperature, similar conditions to the reaction of the initiation-growth experiment. The polymer product $P2VP_{46}$/PhSeBr complex was then precipitated into hexane 3 times before the resulting solid was dried under vacuum. The formation of dynamic covalent bonds was confirmed by nuclear magnetic resonance (^{1}H NMR, Supplementary Fig. 1) and X-ray photoelectron spectroscopy (XPS) analysis (Supplementary Fig. 2). The *Se3d* binding energy of PhSeBr shifts from 56.0 eV to 58.5 eV, and the integration of the peak area corresponds to a conversion degree of 67.6 %; while the *N1s* binding energy of pyridyl groups in P2VP shifts from 389.9 eV to 401.3 eV, and the integration of the peak area corresponds to a conversion degree of 13.6 %. Both results clearly confirmed the existence of the Se-N dynamic covalent bonds[4,5].

General method for the initiation-growth experiment. For initiation-growth experiment, mixture of initiators and $P2VP_{68}$-*b*-$PFMA_{41}$ with R_I and C_P was dispersed in *i*-PrOH and heated at 80 ºC for 20 min before cooling down to 20 ºC. The solution was stirred gently during heating process and was not stirred during cooling.

The mass of the initiation section (M_I) and total mass of the cylinder (M) calculations. We assumed that the initiation section was an ellipsoid without the two ends along the long axis (as show in Supplementary Fig. 11a). Assuming *a* and *b* are long axis and short axis of the ellipse, respectively, according to the elliptic equation ($x^2 / a^2 + y^2 / b^2 = 1$), the volume of the initiation section (V_I) can be calculated by integrating the elliptical section along the long axis (Supplementary equation 1 and 2). The volume of the growth section (V_G) is the summary of two cylinder of equal length, and it's calculated by Supplementary equation 3. Thus, the total volume of the cylinder (V) consists of the volume of the initiation section (V_I) and the volume of the growth section (V_G) (Supplementary equation 4). The values of D_I, D and L are measured directly from the TEM images, and the calculated V_I, V_G and V are listed in Supplementary Table 3 and Table 6. Assuming the density is uniform throughout the whole micelle, the values of M/M_I equals that of V/V_I.

$$V_I = \int_{-x}^{x} \pi y^2 dx \tag{1}$$

$$= 2\int_{0}^{x} \pi b^2 (1 - \frac{x^2}{a^2}) dx$$

$$= 2\int_{0}^{0.5L_I} \pi b (\frac{D_I}{2})^2 (1 - \frac{x^2}{a^2}) dx$$

$$V_I = 2(\pi (\frac{D_I}{2})^2 \frac{L_I}{2} - \frac{\pi (^{L_I}/_2)^2}{a^2} \frac{(^{D_I}/_2)^3}{3}) \tag{2}$$

$$V_G = \pi (\frac{D}{2})^2 (L - L_I) \tag{3}$$

$$V = V_I + V_G \tag{4}$$

Transition cycle experiment initiated by PhSeBr. For a typical interconversion between point C1 to C2, the 0.1 mL of the solution of pre-formed cylindrical micelles (C_p = 0.1 mg/mL, R_I = 5 %, L_n = 480 nm) was diluted to 2.0 mL, and 0.04 mg of $P2VP_{68}$-*b*-$PFMA_{41}$ was added to achieve a final condition of C_p = 0.025 mg/mL, R_I = 1 %. The solution was subsequently heated at 80 °C for 20 min and cooled down to 20 °C naturally. The other interconversion steps were performed in a similar manner.

Growth kinetic experiment at different temperatures. Desired amount of PhSeBr and $P2VP_{68}$-*b*-$PFMA_{41}$ was dispersed in *i*-PrOH and heated at 80 °C for 20 min. Subsequently, the solutions were quenched into a water bath with controlled temperatures. Aliquots of samples (~5 μL) were taken from the solutions after desired period of intervals and prepared for TEM characterizations. The contour lengths of the cylindrical micelles at different time were measured. As shown in Fig. 1f, the micelle length increased sharply with time initially and then reached a plateau. The length at the end of plateau region was assumed to be a 100 % conversion of the polymers in solution, and thus the conversion degree (α) was determined by comparing the micelles lengths with the final micelle length. The concentration of free polymer chain ([P]) at any time can also be determined by the equation

$$[P] = (1-\alpha)[P]_{total} \quad (5)$$

where $[P]_{total}$ is the initial total concentration of polymer in the solution. The value of [P] decreased continuously with t (Supplementary Fig. 6a). A linear relationship was found between 1/[P] and t at different temperatures (Fig. 1g), and then it can be fitted into the second growth kinetic rate equation

$$-\delta[P]/\delta t = k[P]^2 \quad (6)$$

For cylindrical micelles obtained at various C_P and R_I values, the micelle length increased sharply with time initially and reached a plateau as well (Supplementary Fig. 10a). Although various final micelle lengths were obtained at different conditions, their plots of α *versus* t overlap nicely (Supplementary Fig. 10b), suggesting very similar kinetics. Similarly, the value of [P] in these processes can also be determined, and the relative polymer concentration ($[P]/[P]_{total}$) can also be plotted against t (Supplementary Fig. 10c). These overlapping curves, together with the very close values of k obtained from the linear fitting of Avrami-Erofeev function (Supplementary Fig. 10d)[6],

$$G(\alpha) = [-\ln(1-\alpha)]^2 \quad (7)$$

both suggest the growth rate constant k is insensitive to the values of C_P and R_I.

The complexing ability of different initiators with P2VP homopolymer. The complexing abilities of initiators were tested by characterizing the *i*-PrOH solution of the initiators with P2VP homopolymers with ^{1}H NMR at 70 °C (R_I = 20 %). For initiators forming stronger complex with P2VP, the polymer is getting less soluble and thus broader peaks or even disappearance of peaks are expected[7]. As shown in Supplementary Fig. 15, for DTA and PhSeBr, the complexes dissociated at 70 °C and the peaks of P2VP (7.1-7.4 ppm) are clearly visible. Meanwhile, for the case of DMS, the peaks from P2VP partially merged together. For the case of Cu^{2+}, the peaks are even less visible, and especially the peaks around 7.2 ppm disappeared completely.

Thermo-seeded growth process to prepare uniform cylindrical micelles. Cylindrical micelles initiated by Cu^{2+} (R_I = 5 %, C_P = 0.1 mg/mL, L_n = 338 nm, L_w/L_n=1.02) were used as seed micelles, and the solution was diluted to 0.01 mg/mL before use. To a 1 mL of the seed micelle solution, 4, 8, 16 µL of THF solution of $P2VP_{68}$-*b*-$PFMA_{41}$ (5 mg/mL) was added for a unimer/seed = 2, 4 and 8, respectively. The solution was subsequently heated at 80 °C for 20 min and naturally cooling to 20 °C in 3 h.

Preparation of P(*t*BA-*r*-AA). Partial hydrolysis of P*t*BA_{113} homopolymer was achieved in CH_2Cl_2/TFA at v/v = 95/5 using triethylsilane as the cation scavenger and by controlling the hydrolysis time (45 minutes) at 25 °C[8]. The hydrolysis degree of P(*t*BA-*r*-AA) was determined to be 70 % from ^{1}H NMR (Supplementary Fig. 24).

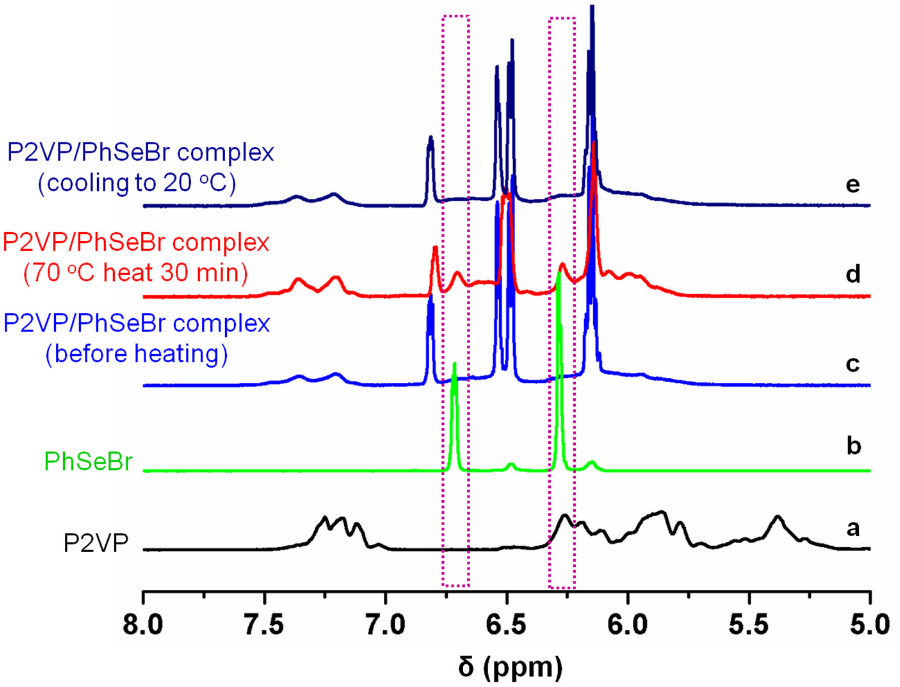

Supplementary Figure 1. ^{1}H NMR spectra of the (a) P2VP homopolymer and (b) PhSeBr at 20 °C, and (c-e) $P2VP_{46}$/PhSeBr complex (c) before heating, (d) heated at 70 °C for 30 min and (e) cooled down back to 20 °C. 2-Propanol-d_8 was used as the solvent.

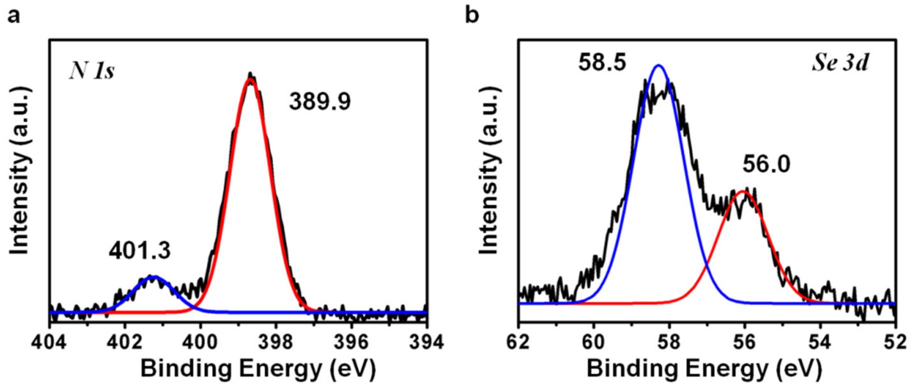

Supplementary Figure 2. XPS spectra of the $P2VP_{46}$/PhSeBr complex. After the formation of dynamic covalent bonds, (a) the *N 1s* binding energy of pyridyl groups in P2VP shifts from 389.9 eV to 401.3 eV, and (b) the *Se 3d* binding energy of PhSeBr shifts from 56.0 eV to 58.5 eV.

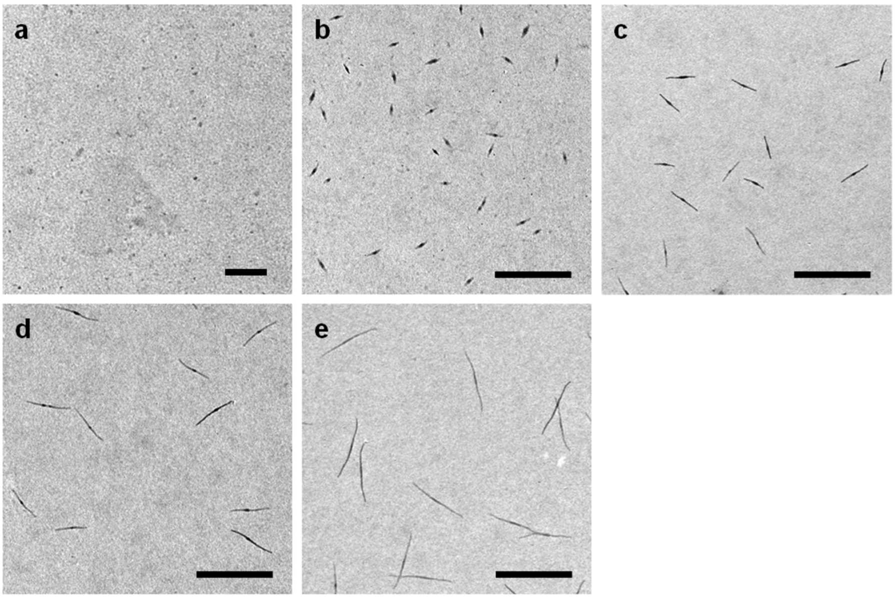

Supplementary Figure 3. TEM images of the cylindrical micelles initiated by PhSeBr (R_I = 5 %, C_P = 0.05 mg/mL). The sample solution was heated at 80 ºC for 20 min (a), and naturally cooled to (b) 70 ºC; (c) 60 ºC; (d) 40 ºC and (e) 20 ºC. Scale bars are 200 nm in image (a) and 1000 nm in images (b-e).

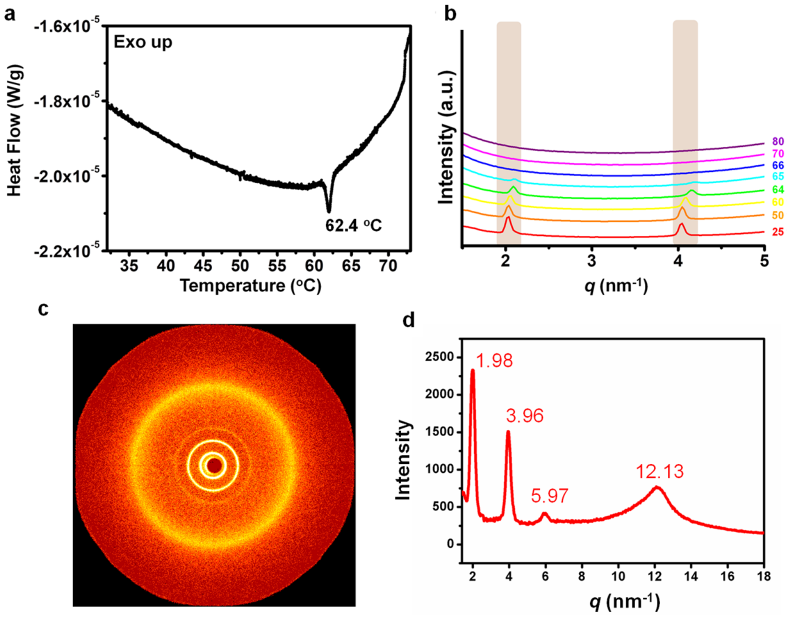

Supplementary Figure 4. (a) High-resolution DSC traces and (b) temperature-dependent in-situ WAXS spectra obtained from the *i*-PrOH solution of $P2VP_{68}$-*b*-$PFMA_{41}$ and PhSeBr. (c) GI-XRD and (d) plot of the scattering intensity along equator *versus* the scattering vector *q* of dried cylindrical micelles (obtained by volatilizing the *i*-PrOH solution of $P2VP_{68}$-*b*-$PFMA_{41}$ diblock copolymer initiated by PhSeBr, C_P = 0.1 mg/mL, R_I = 5 %). The diffraction signals at 1.98, 3.96, 5.97 nm^{-1} at small angles correspond to (001), (002), (003) lamellar planes, and the (001) distance is the interdistance between the lamellar layers. The diffraction signal at 12.13 nm^{-1} at wide angles corresponds to the intermolecular packing distance perpendicular to the local molecular orientation of mesogenic perfluorooctyl moieties.

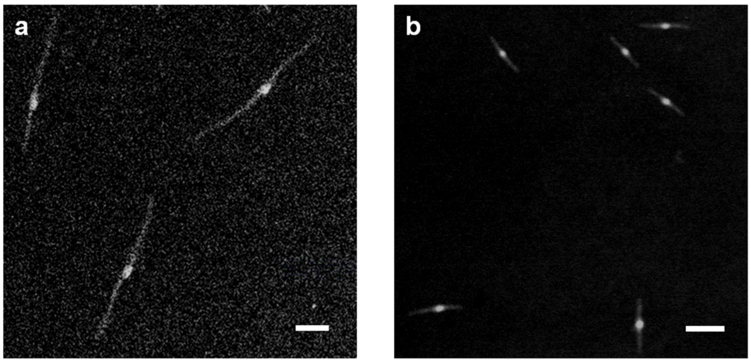

Supplementary Figure 5. Dark field TEM images of the cylindrical micelles initiated by (a) PhSeBr (R_I = 5 %, C_P = 0.05 mg/mL) and (b) Cu^{2+} (R_I = 5 %, C_P = 0.1 mg/mL). Scale bars are 200 nm.

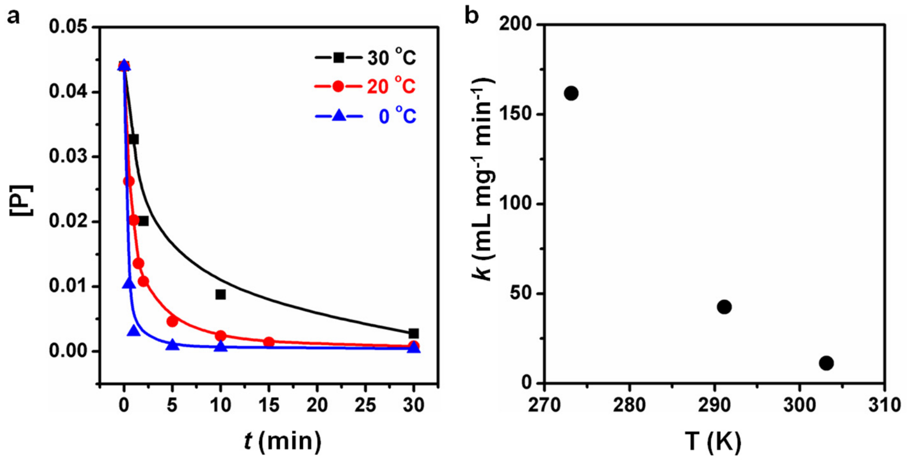

Supplementary Figure 6. (a) The variation of polymer concentration ([P]) *versus* time (*t*) at different temperatures during the initiated assembly process, and (b) the plot of *k versus* temperatures (T).

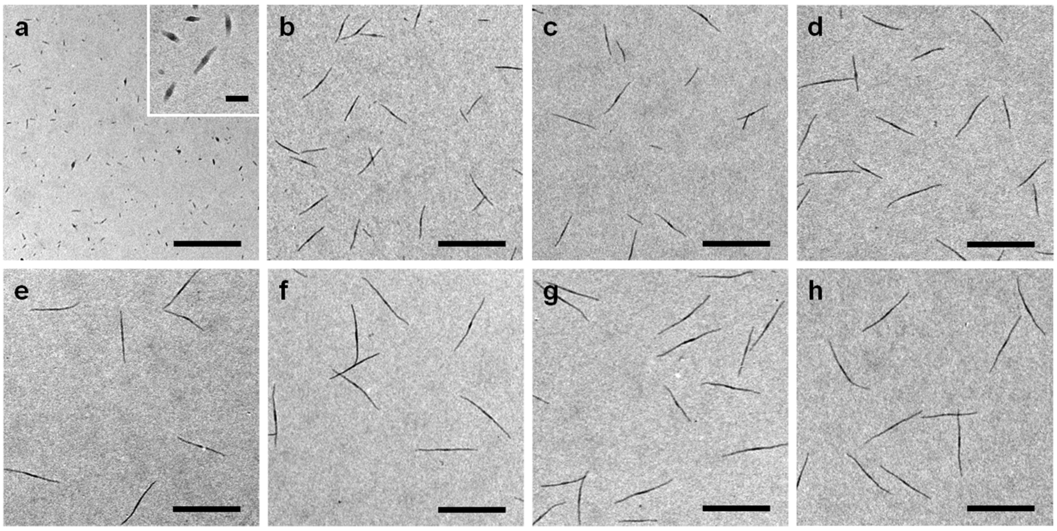

Supplementary Figure 7. TEM images of the samples (R_I = 5 %, C_P = 0.05 mg/mL) obtained by quenching from 80 ºC to 20 ºC (within 15 s) and after a period of (a) 0 s; (b) 30 s; (c) 60 s; (d) 90 s; (e) 2 min; (f) 5 min; (g) 10 min; (h) 15 min. The detailed length information of all the samples is summarized in Supplementary Table 1. Scale bars are 1000 nm, and 100 nm in the inset.

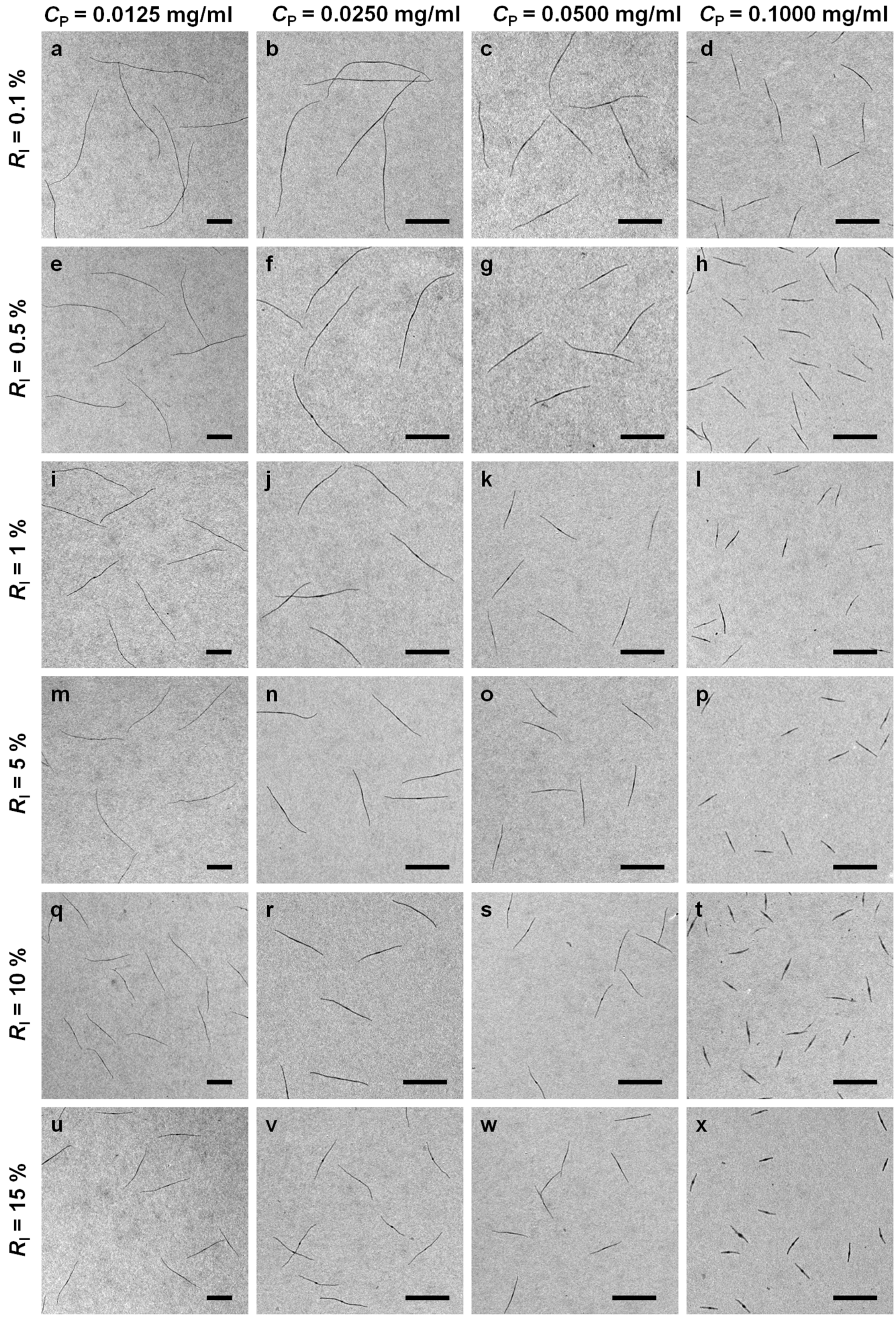

Supplementary Figure 8. TEM images of the cylindrical micelles initiated by PhSeBr with different C_P and R_I. The detailed length information of all the samples is summarized in Supplementary Table 2. Scale bars are 1000 nm.

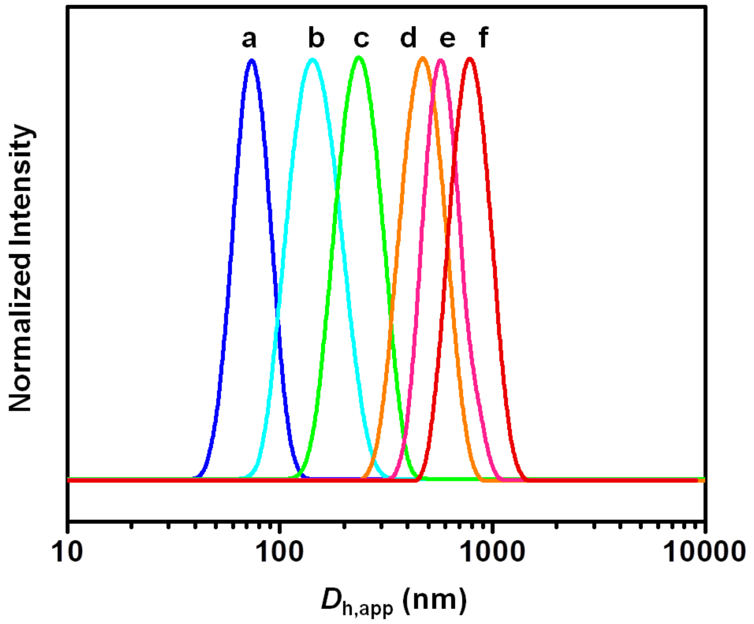

Supplementary Figure 9. The normalized DLS data of the cylindrical samples in *i*-PrOH at 25 °C with different length. (a) L_n = 483 nm, $D_{h,app}$ = 78.9 nm (blue, R_I = 5 %, C_P = 0.1 mg/mL), (b) L_n = 832 nm, $D_{h,app}$ = 164.2 nm (cyan, R_I = 10 %, C_P = 0.05 mg/mL), (c) L_n = 1184 nm, $D_{h,app}$ = 220.2 nm (green, R_I = 1 %, C_P = 0.05 mg/mL), (d) L_n = 2330 nm, $D_{h,app}$ = 458.7 nm (orange, R_I = 0.5 %, C_P = 0.025 mg/mL), (e) L_n = 3040 nm, $D_{h,app}$ = 615.1 nm (pink, R_I = 0.1 %, C_P = 0.025 mg/mL) and (f) L_n = 4576 nm, $D_{h,app}$ =825 nm (red, R_I = 0.1 %, C_P = 0.0125 mg/mL).

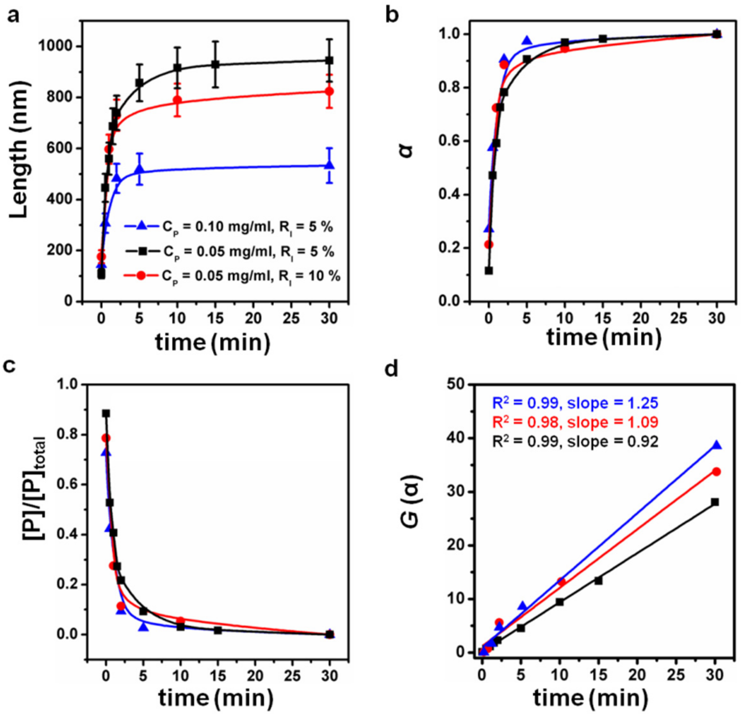

Supplementary Figure 10. The growth kinetics under three different conditions. (a) The variation of L_n *versus* time; (b) the variation of conversion degree (α) *versus* time; (c) the plot of polymer relative concentration ($[P]/[P]_{total}$) *versus* time and (d) linear fitting of Avrami-Erofeev function, $G(\alpha) = [-ln(1-\alpha)]^2$ of the kinetic data. Error bars represent the s.d..

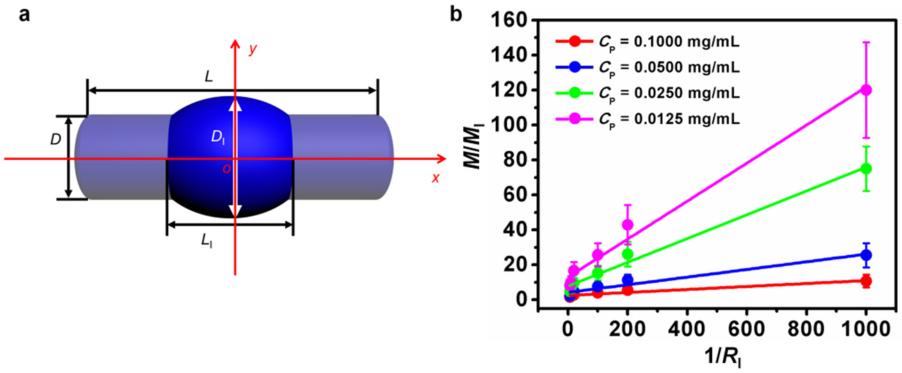

Supplementary Figure 11. (a) The schematic illustration of the initiation section and short cylindrical micelles. The corona chains are removed for clarity. (b) The plots of the (M/M_I) ratio *versus* ($1/R_I$) from the cylindrical micelles initiated by PhSeBr. The detailed length information of all the samples is summarized in Supplementary Table 3. Error bars represent the s.d..

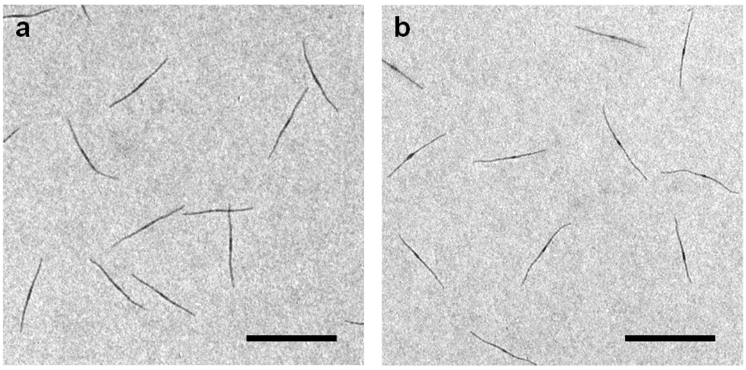

Supplementary Figure 12. TEM images of the cylindrical micelles initiated by PhSeBr (R_I = 5 %, C_P = 0.05 mg/mL). (a) Freshly prepared samples and (b) aged at 20 ^{o}C for over 6 months. Scale bars are 1000 nm.

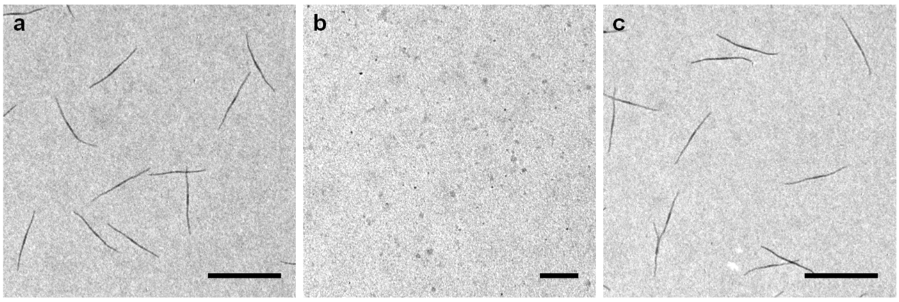

Supplementary Figure 13. TEM image of (a) the cylindrical micelles initiated by PhSeBr (R_I = 5 %, C_P = 0.05 mg/mL, L_n = 974 nm, L_w/L_n = 1.01); then (b) upon heating to 80 ºC for 20 min and (c) naturally cooling to 20 ºC (L_n = 981 nm, L_w/L_n = 1.01). Scale bars are 1000 nm in images (a) and (c), and 200 nm in image (b).

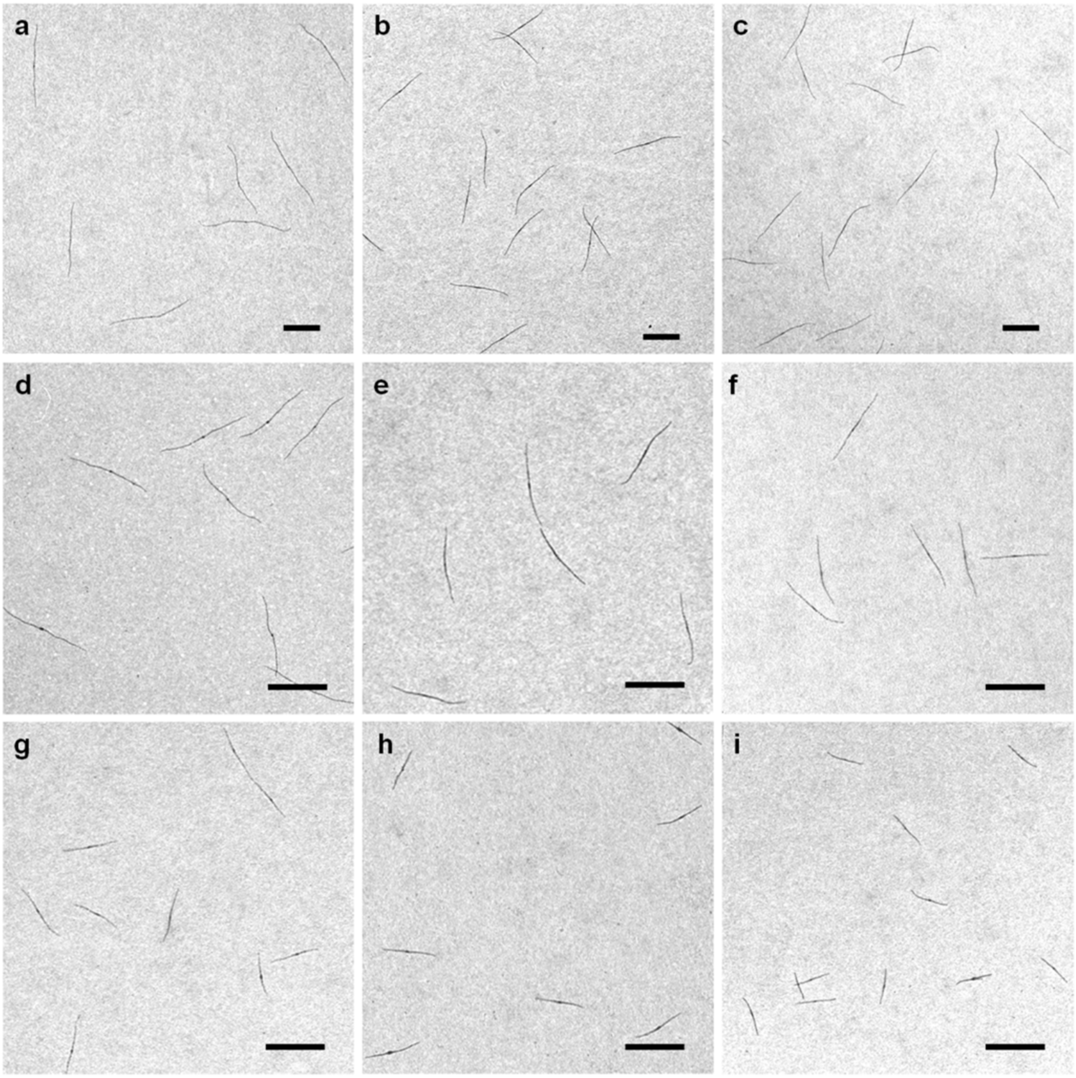

Supplementary Figure 14. TEM images of the linear cylindrical micelles initiated by (a) DLA, (b) DTA, (c) BC, (d) PFIB, (e) PhSeBr, (f) DMS, (g) DIFB, (h) Pt(0) and (i) Cu^{2+}, respectively. R_I = 5 %, C_P = 0.025 mg/mL in all samples. The detailed length information of all the samples is summarized in Supplementary Table 4. Scale bars are 1000 nm.

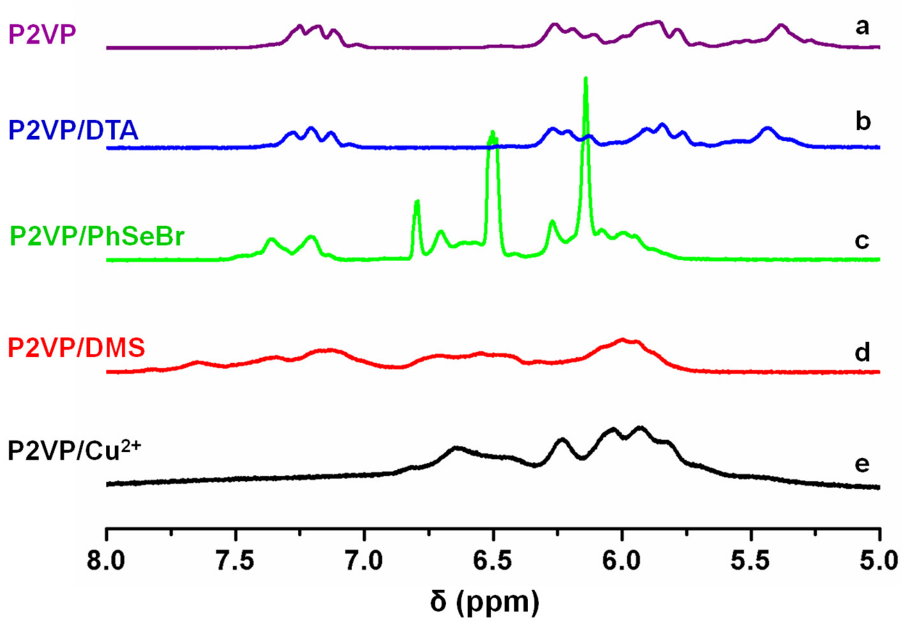

Supplementary Figure 15. ^{1}H NMR spectra of (a) $P2VP_{46}$; (b) $P2VP_{46}$/DTA complex; (c) $P2VP_{46}$ / PhSeBr complex; (d) $P2VP_{46}$/DMS complex and (e) $P2VP_{46}/Cu^{2+}$ complex heated at 70 ºC for 30 min. 2-Propanol-d_8 was used as the solvent.

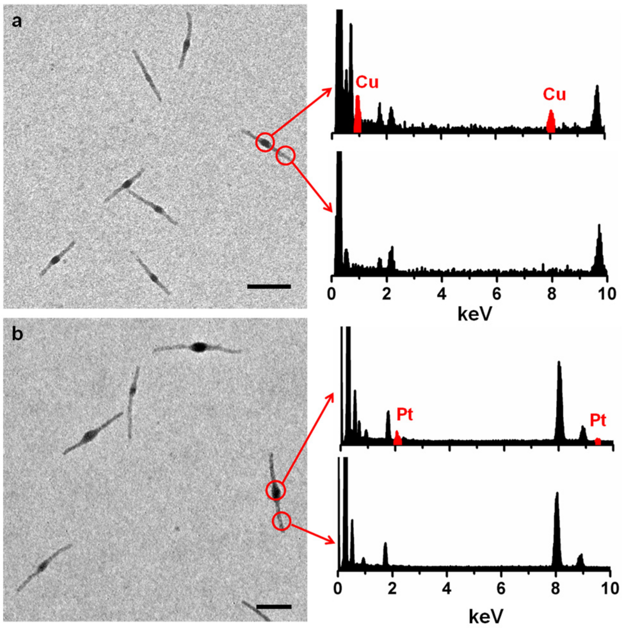

Supplementary Figure 16. TEM images and EDX spot analysis (the location of an EDX spot analysis is labeled by the red circle with a spot diameter of 100 nm) of (a) cylindrical micelles initiated by Cu^{2+} (R_I = 5 %, C_P = 0.1 mg/mL), the EDX lines at ca. 2.1 and 9.7 keV are Au peaks from the Au grid substrate; and (b) cylindrical micelles initiated by Pt(0) (R_I = 5 %, C_P = 0.1 mg/mL), the EDX lines at ca. 8.0 and 9.0 keV are copper peaks from the copper grid substrate. Scale bars are 200 nm.

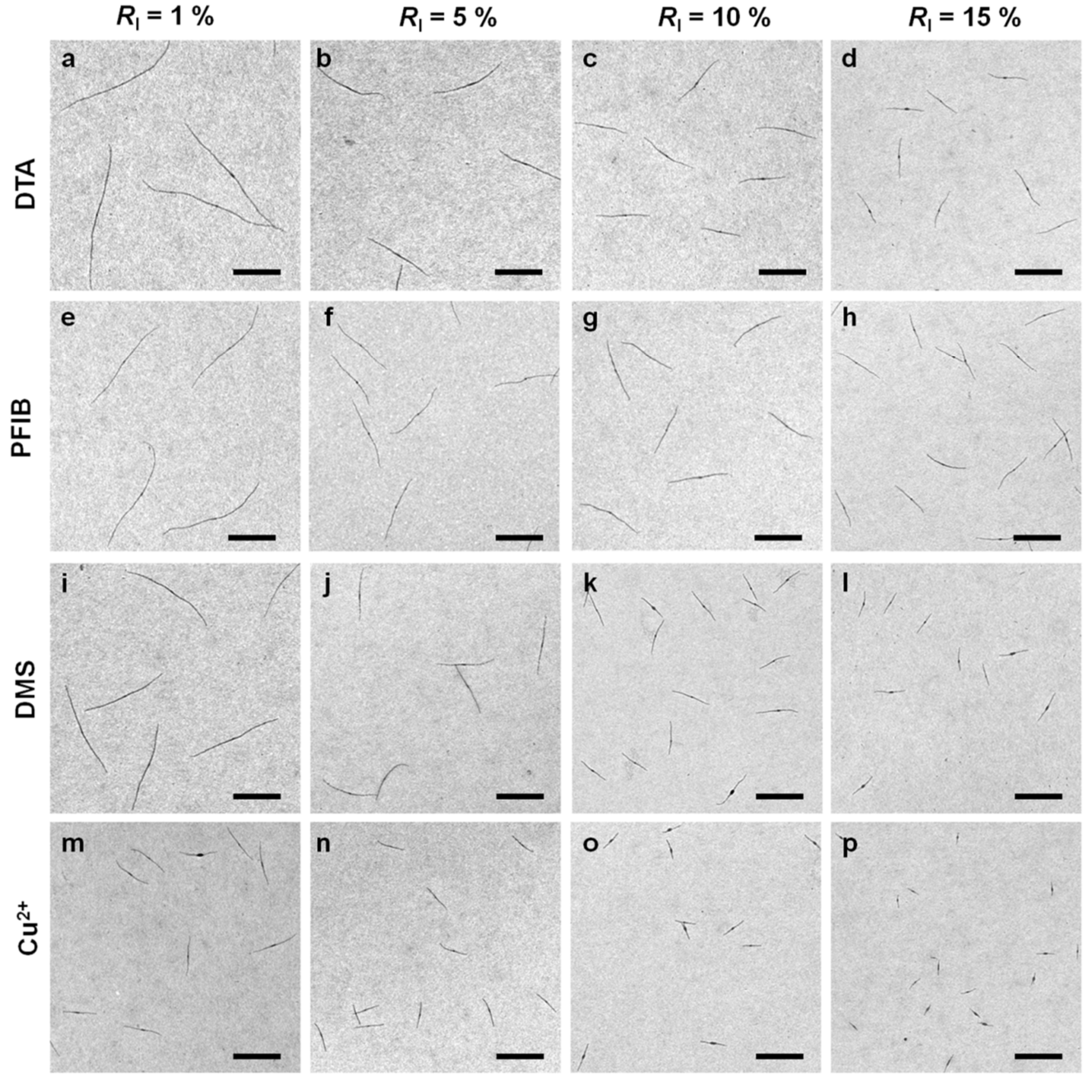

Supplementary Figure 17. TEM images of the linear cylindrical micelles initiated by small molecule initiators with a fixed C_P of 0.025 mg/mL. (a-d) initiated by DTA, (e-h) by PFIB, (i-l) by DMS, (m-p) by Cu^{2+}, (a, e, i, m) R_I = 1 %; (b, f, j, n) R_I = 5 %; (c, g, k, o) R_I = 10 %; (d, h, l, p) R_I = 15 %. The detailed length information of all the samples is summarized in Supplementary Table 5. Scale bars are 1000 nm.

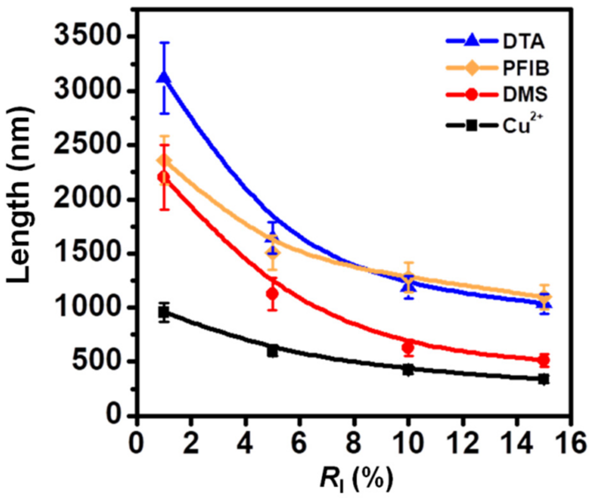

Supplementary Figure 18. The variation of cylindrical micelle length *versus* R_I initiated by small molecule initiators with a fixed C_P of 0.025 mg/mL. (blue, initiated by DTA; orange, initiated by PFIB; red, initiated by DMS; black, initiated by Cu^{2+}). The detailed length information of all the samples is summarized in Table S5. Error bars represent the s.d..

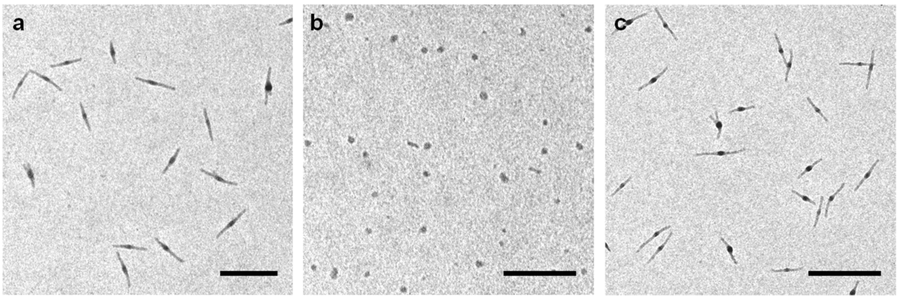

Supplementary Figure 19. TEM image of (a) the cylindrical micelles initiated by Cu^{2+} (R_I = 5 %, C_P = 0.1 mg/mL); then diluted to C_P = 0.025 mg/mL and (b) upon heating to 80 ºC for 20 min and (c) naturally cooling to 20 ºC. The detailed length information of all the samples is summarized in Supplementary Table 7. Scale bars are 500 nm.

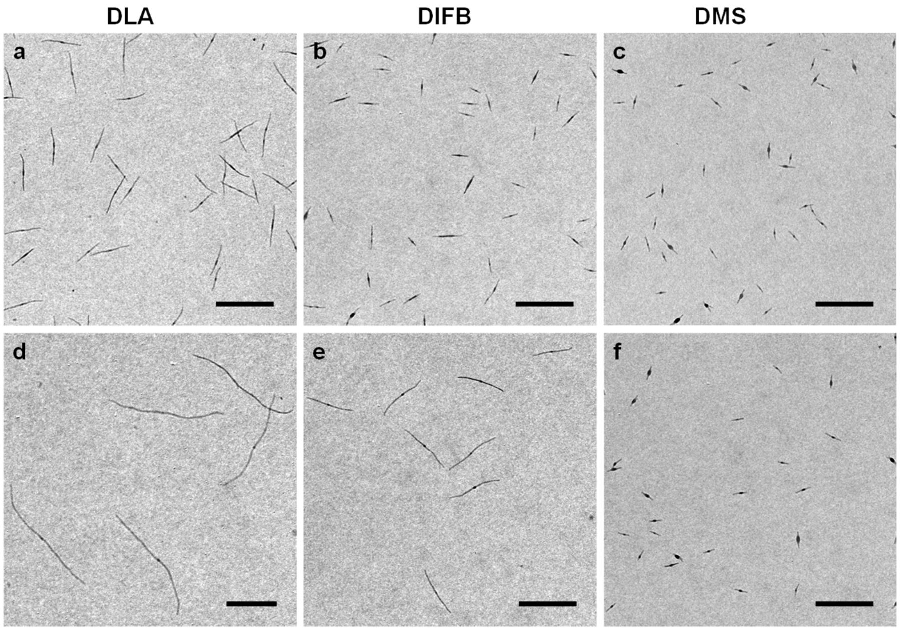

Supplementary Figure 20. TEM image of the cylindrical micelles initiated by small molecule initiators with fixed C_P = 0.1 mg/mL, R_I = 5 %, and initiated by (a) DLA, (b) DIFB and (c) DMS, respectively. The upper row: the original cylindrical micelles; the lower row: the solutions were diluted to C_P = 0.025 mg/mL and subsequently heated to 80 ºC for 20 min and cooled naturally to 20 ºC. The detailed length information of all the samples is summarized in Supplementary Table 7. Scale bars are 1000 nm.

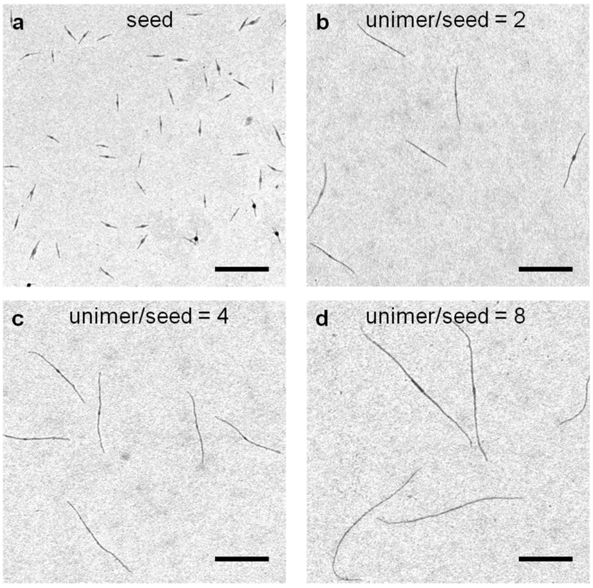

Supplementary Figure 21. TEM images of the cylindrical micelles produced from the thermo-seeded growth process. (a) seed micelles; (b-d) produced from the a unimer-to-seed ratio (unimer/seed) of (b) 2; (c) 4; and (d) 8. The detailed length information of all the samples is summarized in Supplementary Table 8. Scale bars are 1000 nm.

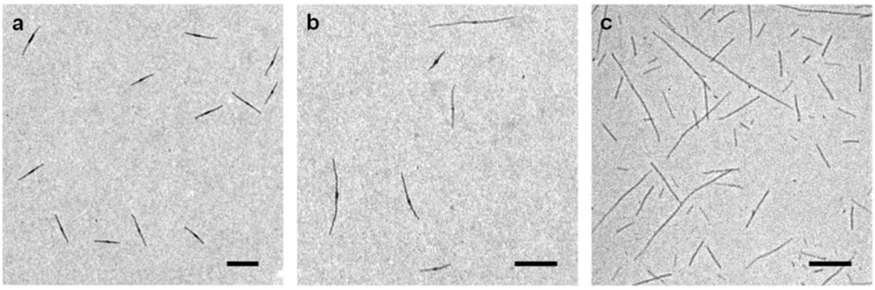

Supplementary Figure 22. TEM images of (a) the cylindrical micelles initiated by PhSeBr (R_I = 5 %, C_P = 0.1 mg/mL); (b) the cylindrical micelles obtained by adding equimolar amount of 4-dimethylaminopyridine (DMAP) and heating to 80 °C for 20 min, then naturally cooling to 20 °C; and (c) the cylindrical micelles formed by dispersing the P2VP$_{68}$-*b*-PFMA$_{41}$ diblock copolymer in *i*-PrOH without initiators (C_P = 0.1 mg/mL). Scale bars are 500 nm.

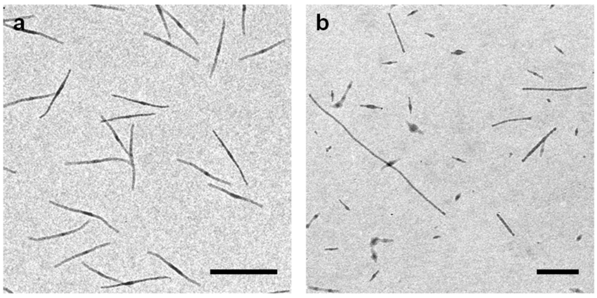

Supplementary Figure 23. TEM images of (a) the cylindrical micelles initiated by Cu^{2+} (R_I = 5 %, C_P = 0.025 mg/mL); (b) the cylindrical micelles obtained by adding equimolar amount of N,N,N',N",N"-pentamethyldiethylenetriamine (PMDETA) and heating to 80 ºC for 20 min, then naturally cooling to 20 ºC. Scale bars are 500 nm.

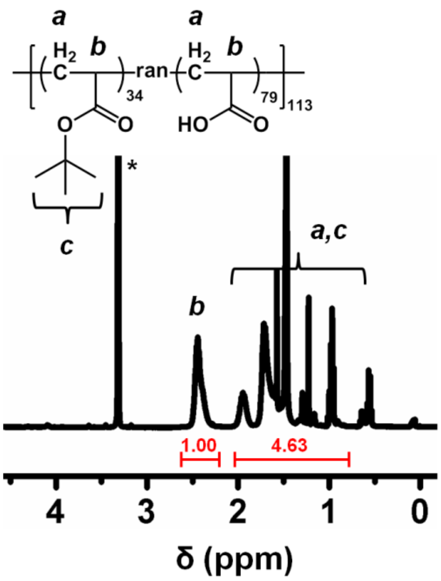

Supplementary Figure 24. ^{1}H NMR spectrum of the P(*t*BA-*r*-AA)$_{113}$ random copolymer. CD_3OD was used as the solvent.

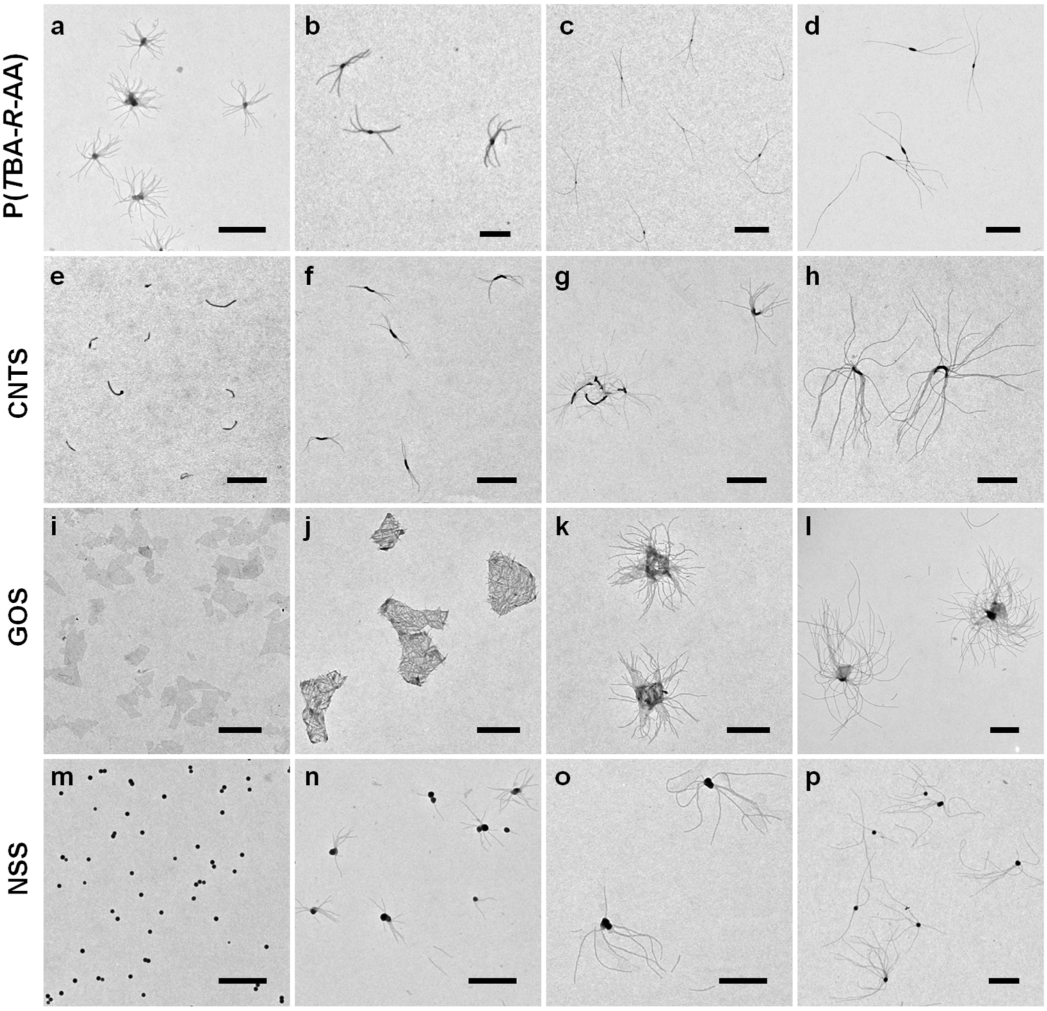

Supplementary Figure 25. TEM images of the different hierarchical structures initiated by large initiators. (a-d) initiated by P(*t*BA-*r*-AA)$_{113}$, (a) R_I = 10 wt.%, C_P = 0.05 mg/mL; (b) R_I = 2 wt.%, C_P = 0.05 mg/mL; (c) R_I = 5 wt.%, C_P = 0.025 mg/mL and (d) R_I = 2 wt.%, C_P = 0.025 mg/mL. (e-h) initiated by CNTs, (e) CNTs; (f) R_I = 10 wt.%, C_P = 0.025 mg/mL; (g) R_I = 2 wt.%, C_P = 0.05 mg/mL and (h) R_I = 2 wt.%, C_P = 0.025 mg/mL. (i-l) initiated by GOs, (i) GOs; (j) R_I = 5 wt.%, C_P = 0.05 mg/mL; (k) R_I = 2 wt.%, C_P = 0.05 mg/mL and (l) R_I = 2 wt.%, C_P = 0.025 mg/mL. (m-p) initiated by NS, (m) NS; (n) R_I = 5 wt.%, C_P = 0.025 mg/mL; (o) R_I = 5 wt.%, C_P = 0.05 mg/mL and (p) R_I = 2 wt.%, C_P = 0.025 mg/mL. The detailed length information of all the samples is summarized in Supplementary Table 9. Scale bars are 1000 nm.

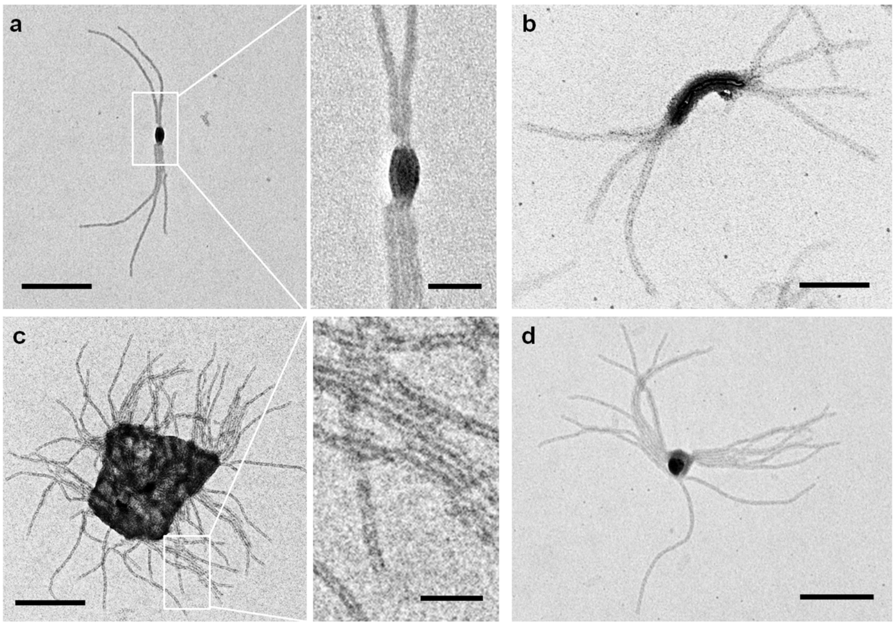

Supplementary Figure 26. TEM images of RuO_4-stained hierarchical structures initiated by large initiators. (a) P(*t*BA-*r*-AA) (R_I = 2 wt.%, C_P = 0.05 mg/mL); (b) CNTs (R_I = 10 wt.%, C_P = 0.025 mg/mL); (c) GOs (R_I = 2 wt.%; C_P = 0.05 mg/mL) and (d) NS (R_I = 2 wt.%; C_P = 0.025 mg/mL). Scale bars are 500 nm in images (a), (c) and (d), 200 nm in image (b), and 100 nm in the enlarged images.

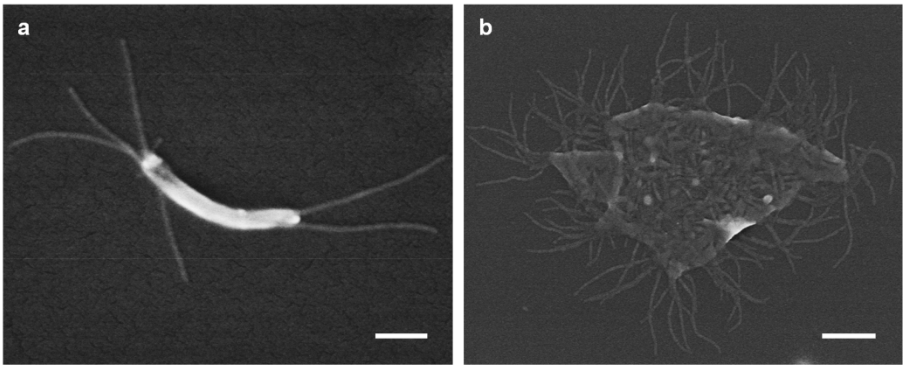

Supplementary Figure 27. SEM images of the hierarchical structures initiated by large initiators. (a) CNTs (R_I = 10 wt.%, C_P = 0.025 mg/mL) and (b) GOs (R_I = 2 wt.%; C_P = 0.05 mg/mL). Scale bars are 200 nm in image (a), and 500 nm in image (b).

Supplementary Table 1. Values of L_n, L_w, L_w/L_n, σ and σ/L_n of cylindrical micelles obtained by quick quenching from 80 ºC to 20 ºC (within 15 s) at different growth time (C_P = 0.05 mg/mL, R_I = 5 % PhSeBr)

**Samples**	**L_n (nm)**	**L_w (nm)**	**L_w/L_n**	**σ (nm)**	**σ/L_n**
**0 s**	109	112	1.03	18	0.165
**30 s**	446	453	1.01	55	0.123
**60 s**	560	567	1.01	63	0.113
**90 s**	687	694	1.01	70	0.102
**2 min**	740	745	1.01	67	0.091
**5 min**	857	867	1.01	72	0.084
**10 min**	916	923	1.01	80	0.087
**15 min**	929	938	1.01	90	0.097

Supplementary Table 2. Values of L_n, L_w, L_w/L_n, σ and σ/L_n of cylindrical micelles initiated by PhSeBr with different C_p and R_I

C_P (mg/mL)	R_I (%)	L_n (nm)	L_w (nm)	L_w/L_n	σ (nm)	σ/L_n
**0.1000**	0.1	765	780	1.02	108	0.141
	0.5	643	654	1.02	93	0.145
	1.0	563	571	1.01	66	0.117
	5.0	483	489	1.01	51	0.106
	10.0	431	439	1.02	59	0.138
	15.0	374	380	1.02	46	0.123
**0.0500**	0.1	1699	1714	1.01	169	0.099
	0.5	1383	1400	1.01	152	0.110
	1.0	1184	1200	1.01	139	0.117
	5.0	974	986	1.01	107	0.110
	10.0	832	843	1.01	97	0.117
	15.0	703	714	1.02	78	0.111
**0.0250**	0.1	3040	3066	1.01	284	0.093
	0.5	2330	2351	1.01	201	0.086
	1.0	1704	1723	1.01	182	0.107
	5.0	1415	1425	1.01	121	0.086
	10.0	1137	1147	1.01	109	0.096
	15.0	1024	1034	1.01	99	0.097
**0.0125**	0.1	4576	4603	1.01	346	0.076
	0.5	3625	3644	1.01	294	0.081
	1.0	2904	2939	1.01	254	0.087
	5.0	2486	2521	1.01	224	0.090
	10.0	2064	2084	1.01	205	0.099
	15.0	1690	1718	1.02	182	0.108

Supplementary Table 3. Values of L_I, D_I, V_I, V and V/V_I of cylindrical micelles initiated by PhSeBr with different C_P and R_I (D is 22.6 nm)*

C_P (mg/mL)	R_I (%)	L_I (nm)	D_I (nm)	V_I (nm^3)	V (nm^3)	V/V_I
**0.1000**	0.1	58.7	31.2	43381.5	420203.1	9.7
	0.5	79.0	34.7	68369.2	373523	5.5
	1.0	85.2	37.8	90101.9	349722.5	3.9
	5.0	91.5	43.5	126884.1	338923.7	2.7
	10.0	100.4	50.9	184008.8	365926.4	2.0
	15.0	107.8	56.7	259371.1	409226.8	1.6
**0.0500**	0.1	55.7	31.5	43379.9	928881	21.4
	0.5	76.7	33.7	68369.2	772276	11.3
	1.0	81.2	37.6	90101.9	684351.7	7.6
	5.0	89.5	42.5	126884.1	603501.7	4.8
	10.0	98.4	50.1	184008.8	582007.4	3.2
	15.0	104.9	58.7	259371.1	586510.2	2.3
**0.0250**	0.1	50.2	31.5	35321.1	1646499	46.6
	0.5	60.8	29.6	44675.4	1267877	28.4
	1.0	67.2	31.1	50104.8	932749.9	18.6
	5.0	89.0	36.3	76590.9	791652.1	10.3
	10.0	93.5	38.9	94075.1	656640.1	7.0
	15.0	98.9	41.7	122252.6	617999.6	5.1
**0.0125**	0.1	45.3	30.0	29903.6	2471296	82.6
	0.5	50.8	30.8	37958.2	1964366	51.8
	1.0	60.2	31.1	62055.6	1594560	25.7
	5.0	65.3	33.3	78389.1	1382419	17.6
	10.0	88.2	35.9	120106.6	1184885	9.9
	15.0	95.8	38.7	152265	191709.2	1.3

*: Assuming density is uniform inside the cylindrical micelles, and thus $M/M_I=V/V_I$.

Supplementary Table 4. Values of L_n, L_w, L_w/L_n, σ and σ/L_n of micelles initiated by small molecules at C_p = 0.025 mg/mL and R_I = 5 %

**Small molecule initiators**	**L_n (nm)**	**L_w (nm)**	**L_w/L_n**	**σ (nm)**	**σ/L_n**
**DLA**	2138	2149	1.01	157	0.073
**DTA**	1644	1658	1.01	144	0.088
**BC**	1578	1601	1.01	184	0.117
**PFIB**	1505	1521	1.01	159	0.106
**PhSeBr**	1415	1425	1.01	121	0.086
**DMS**	1241	1263	1.02	150	0.121
**DIFB**	937	946	1.01	91	0.097
**Pt(0)**	819	827	1.01	81	0.099
**Cu^{2+}**	600	605	1.01	50	0.083

Supplementary Table 5. Values of L_n, L_w, L_w/L_n, σ and σ/L_n of micelles initiated by DTA, PFIB, DMS and Cu^{2+} at C_p = 0.025 mg/mL with different R_I

**Small-molecule initiators**	R_I **(%)**	L_n **(nm)**	L_w **(nm)**	L_w/L_n	**σ (nm)**	**σ/**L_n
**DTA**	1.0	3120	3151	1.01	325	0.104
	5.0	1644	1658	1.01	144	0.088
	10.0	1190	1204	1.01	104	0.088
	15.0	1037	1046	1.01	90	0.087
**PFIB**	1.0	2360	2525	1.01	219	0.093
	5.0	1505	1521	1.01	159	0.106
	10.0	1260	1330	1.01	139	0.110
	15.0	1098	1197	1.01	112	0.102
**DMS**	1.0	2203	2249	1.02	300	0.136
	5.0	1241	1263	1.02	150	0.121
	10.0	689	694	1.01	75	0.109
	15.0	510	516	1.01	60	0.118
**Cu^{2+}**	1.0	889	899	1.01	88	0.099
	5.0	600	605	1.01	50	0.083
	10.0	427	432	1.01	35	0.082
	15.0	325	330	1.01	32	0.098

Supplementary Table 6. Values of L_I, D_I, V_I, V and V/V_I of cylindrical micelles initiated by DTA, PFIB, DMS and Cu^{2+} at C_P = 0.025 mg/mL with different R_I (D of cylinder is 22.6 nm)*

**Small-molecule initiators**	R_I **(%)**	L_I **(nm)**	D_I **(nm)**	V_I **(nm^3)**	V **(nm^3)**	V/V_I
**DTA**	1.0	53	33.4	43668.5	1696338	38.8
	5.0	70	36.4	72795.3	920953.7	12.7
	10.0	74	40.2	93861.6	695224.2	7.4
	15.0	84	44	127641.8	641171	5.0
**PFIB**	1.0	53	34.1	48538.3	1208602	24.9
	5.0	63	37.5	83002.1	846621.3	10.2
	10.0	73	42.1	116959.9	783631.3	6.7
	15.0	86	49.7	165724.8	712616.7	4.3
**DMS**	1.0	54	34	38143.7	1196144	31.4
	5.0	74	39.1	88794.9	717639.2	8.1
	10.0	77	49.5	148086.9	477866.4	3.2
	15.0	86	55.1	204938.8	433413.5	2.1
**Cu^{2+}**	1.0	54	37.6	59920	509864.3	8.5
	5.0	89	41.6	120887.7	396242.8	3.3
	10.0	91	43.8	137024.3	318079.7	2.3
	15.0	95	48.1	172515.2	303457.1	1.8

*: Assuming density is uniform inside the cylindrical micelles, and thus $M/M_I=V/V_I$.

Supplementary Table 7. Values of L_n, L_w, L_w/L_n, σ and σ/L_n of micelles initiated by DLA, DIFB, DMS and Cu^{2+} at C_p = 0. 1 mg/mL, R_I = 5 %, and then diluted to C_P = 0.025 mg/mL and upon heating to 80 °C for 20 min and naturally cooling to 20 °C

**Small-molecule initiators**	**C_p (mg/mL)**	**L_n (nm)**	**L_w (nm)**	**L_w/L_n**	**σ (nm)**	**σ/L_n**
**DLA**	0.100	636	646	1.02	77	0.121
	0.025	2249	2263	1.01	178	0.079
**DIFB**	0.100	325	330	1.02	32	0.098
	0.025	927	936	1.01	89	0.096
**DMS**	0.100	274	278	1.02	29	0.106
	0.025	271	274	1.01	28	0.103
**Cu^{2+}**	0.100	338	345	1.02	32	0.095
	0.025	341	345	1.01	31	0.091

Supplementary Table 8. Values of L_n, L_w, L_w/L_n, σ and σ/L_n of micelles produced from the thermo-seeded growth process

unimer/seed	L_n (nm)	L_w (nm)	L_w/L_n	σ (nm)	σ/L_n
**seed**	338	345	1.02	32	0.095
**2**	933	947	1.02	113	0.121
**4**	1602	1617	1.01	157	0.098
**8**	3114	3147	1.01	308	0.099

Supplementary Table 9. Values of L_n, L_w, L_w/L_n, σ and σ/L_n of micelles initiated by CNTs and NSs with different C_p and R_I

**Initiators**	**R_I (wt. %), C_P (mg/mL)**	**L_n (nm)**	**L_w (nm)**	**L_w/L_n**	**σ (nm)**	**σ/L_n**	**$\bar{N}_b$**
**CNTs**	10 %, 0.025	541	554	1.02	85	0.157	7
	2 %, 0.050	1017	1040	1.02	154	0.152	11
	2 %, 0.025	2394	2433	1.02	307	0.128	20
**NSs**	5 %, 0.025	501	509	1.02	64	0.129	5
	2 %, 0.050	1034	1052	1.02	136	0.131	8
	2 %, 0.025	2243	2293	1.02	321	0.143	12

References

1 Wang, S., Tozaki, K., Hayashi, H. & Inaba, H. Nano-watt stabilized DSC and ITS applications. *J. Therm. Anal. Calorim.* **79**, 605-613, (2005).

2 Ema, K. & Yao, H. Some aspects of recent improvements of temperature-modulated calorimeter. *Thermochim. Acta* **305**, 157-163, (1997).

3 Aya, S. *et al.* Observation of Two Isotropic-Nematic Phase Transitions Near a Surface. *Phys. Rev. Lett.* **106**, 117801, (2011).

4 Yi, Y. *et al.* Fabrication of well-defined crystalline azacalixarene nanosheets assisted by Se center dot center dot center dot N non-covalent interactions. *Chem. Commun.* **48**, 7495-7497, (2012).

5 Yi, Y., Xu, H., Wang, L., Cao, W. & Zhang, X. A New Dynamic Covalent Bond of SeN: Towards Controlled Self-Assembly and Disassembly. *Chem. Eur. J.* **19**, 9506-9510, (2013).

6 Debruijn, T. J. W., Dejong, W. A. & Vandenberg, P. J. Kinetic-parameters in Avrami-Erofeew type reactions from isothermal and non-isothermal experiments. *Thermochim. Acta* **45**, 315-325, (1981).

7 Li, X., Liu, G. & Han, D. Wrapping amino-bearing block copolymer cylinders around carboxyl-bearing nanofibers: a case of hierarchical assembly. *Soft Matter* **7**, 8216-8223, (2011).

8 Zheng, R., Wang, J., Liu, G. & Jao, T.-C. Lubricant-oil-dispersible stainless-steel-binding block copolymer nanoaggregates and nanospheres. *Macromolecules* **40**, 7601-7608, (2007).
